# A Semi-Supervised Learning Algorithm for Predicting Four Types MiRNA-Disease Associations by Mutual Information in a Heterogeneous Network

**DOI:** 10.3390/genes9030139

**Published:** 2018-03-02

**Authors:** Xiaotian Zhang, Jian Yin, Xu Zhang

**Affiliations:** School of Mechanical, Electrical and Information Engineering, Shandong University, Weihai 264209, China; zhangxiaotian@mail.sdu.edu.cn (Xiaotian.Z.); yunkaiyibi@163.com (Xu.Z.)

**Keywords:** multiple type miRNA-disease association prediction, semi-supervised learning, network similarity, label propagation algorithm

## Abstract

Increasing evidence suggests that dysregulation of microRNAs (miRNAs) may lead to a variety of diseases. Therefore, identifying disease-related miRNAs is a crucial problem. Currently, many computational approaches have been proposed to predict binary miRNA-disease associations. In this study, in order to predict underlying miRNA-disease association types, a semi-supervised model called the network-based label propagation algorithm is proposed to infer multiple types of miRNA-disease associations (NLPMMDA) by mutual information derived from the heterogeneous network. The NLPMMDA method integrates disease semantic similarity, miRNA functional similarity, and Gaussian interaction profile kernel similarity information of miRNAs and diseases to construct a heterogeneous network. NLPMMDA is a semi-supervised model which does not require verified negative samples. Leave-one-out cross validation (LOOCV) was implemented for four known types of miRNA-disease associations and demonstrated the reliable performance of our method. Moreover, case studies of lung cancer and breast cancer confirmed effective performance of NLPMMDA to predict novel miRNA-disease associations and their association types.

## 1. Introduction

MicroRNAs (miRNAs) are small endogenous non-coding RNAs that mainly regulate gene expression at the post-transcriptional level [1,2,3]. They are evolutionarily conserved and play a regulatory role by base pairing with messenger RNAs (mRNAs), resulting in mRNA degradation or translation inhibition [2,4,5]. Increasing evidence suggests that miRNAs are involved in a variety of critical biological processes, such as development, differentiation, apoptosis and metabolism [2]. Since the discovery of lin-4 and let-7 [6,7], many researchers have focused on the study of miRNAs, and numerous miRNAs have been identified. Furthermore, a great deal of databases have been established to provide information on miRNAs, such as the Human microRNA Disease Database (HMDD) [8], miR2Disease [9], database of Differentially Expressed miRNAs in human Cancers (dbDEMC) [10] and so on. It has been demonstrated that dysregulation of miRNAs may lead to a variety of diseases [11,12,13]. For example, miR-21 can target the *MAP2K3* gene directly during the carcinogenesis of hepatocellular carcinoma, resulting in expression inhibition of *MAP2K3* [14]. This also indicates that miRNAs can serve as efficient biomarkers for disease detection, diagnosis and prognosis [15]. Therefore, identifying disease-related miRNAs is a crucial problem.

During the past few decades, various disease-related miRNAs have been identified by several experimental methods. However, with the increasing of new miRNAs and other biological information, these experimental methods face severe challenges, such as long experimental periods and expensive equipment. Thus, many computational approaches have been proposed to predict miRNA-disease associations [16]. The goal of computational approaches is to reduce the number of candidate miRNAs. Based on abundant biological information, several network-based methods are proposed to infer the relationships between miRNAs and diseases. The key of network-based methods is to calculate similarity scores among miRNAs and diseases over networks. It is well known that miRNAs with similar functions tend to be associated with similar diseases and vice versa [16,17]. Based on this assumption, Wang et al. [18] provided a method to infer human miRNA functional similarity (MISIM) by measuring semantic similarity of diseases which associated with miRNAs. Furthermore, they constructed a miRNA functional network. On the basis of results studied in [18], Xuan et al. [19] calculated miRNA functional similarity by integrating information of disease terms and similarity of disease phenotype. A new method was proposed to predict disease-related miRNAs, which is based on weighted *k* of most similar neighbors. The weights were assigned by miRNA family or cluster information. This method exhibited a good performance, but it was not applicable to diseases without any known associated miRNAs. Therefore, Chen and Zhang [20] adopted a network-consistency-based inference method to predict potential disease-related miRNAs based on the data derived from the miRNA functional similarity network, disease phenotype similarity network, and known miRNA-disease interaction network, which can be applied to isolated diseases without any related miRNAs. Nevertheless, the performance is not particularly satisfactory. Recently, Gu et al. [21] proposed a global and effective method to infer the associations between miRNAs and diseases, which is called network consistency projection for miRNA-disease associations (NCPMDA). NCPMDA is a non-parametric approach and takes full advantage of various molecular data, including miRNA functional similarity network, disease semantic similarity network, validated known miRNA-disease associations and miRNA family information. NCPMDA is applicable to isolated diseases and the predictive performance is superior to the previous method. The main problem of the above algorithms is that they all adopted similarity scores calculated by Wang et al. [18] that are estimated by miRNA-related diseases. However, the related disease information of many miRNAs remains largely unknown. In addition, these methods do not allow identification of the underlying proteins involved in miRNA-disease associations. In order to reveal the underlying proteins, Mørk et al. [22] developed a protein-driven method, miRPD, to infer miRNA-disease associations by using miRNA-protein associations and protein-disease associations. Without using any known information of miRNA-disease relationships, this method measured the associations of miRNAs and diseases via calculating association scores of miRNA-protein and protein-disease. The miRPD method attempted to reveal the underlying proteins involved in miRNA-disease associations and exhibited a reliable result.

Machine-learning-based algorithms are beneficial to improve the prediction performance, and up to now, several studies have proposed machine-learning-based models to predict potential miRNA-disease associations. For example, Jiang et al. [23] trained a support vector machine classifier by a feature vector to distinguish positive miRNA-disease associations from large-scale negative ones. Xu et al. [24] constructed a support vector machine classifier based on the features and changes in miRNA expression; then, the classifier was applied to an miRNA target–dysregulated network to infer new disease miRNAs. Generally, the common limitation of these methods is the selection of negative samples. It is known that there are no experimental validated negative associations between miRNAs and diseases, and miRNA-disease pairs selected from unknown miRNA-disease pairs might appear to be too biased. Considering this fact, Chen and Yan [25] proposed a semi-supervised method motivated by the framework of regularized least squares to infer potential miRNA-disease associations (RLSMDA). RLSMDA exhibited a reliable performance by leave-one-out cross validation (LOOCV) and case studies. In addition, it can work for diseases without any known related miRNAs. However, it might have biased the results, i.e., that they constructed disease similarity network and miRNA similarity network only by disease semantic similarity and miRNA functional similarity, respectively. Recently, based on the hypothesis that distributional semantics can reveal information of relationships between miRNAs and diseases, Pasquier and Gardès [26] proposed a vector space model to discover new disease-miRNA associations. In this method, distributional information of miRNAs and diseases is represented in a high-dimensional vector space, which contains miRNA-disease associations, miRNA-related target mRNAs, family information of miRNAs, and genomic location information of miRNAs and abstracts of associated studies. By reducing the dimensionality of this high-dimensional vector space to fewer dimensions, they calculated the cosine distance of two vectors to measure their correlations. This method makes full use of miRNA-related information and achieves a satisfactory performance.

All of the above computational methods have identified various novel miRNA-disease interactions, but the specific types of interaction have not been predicted. Thus, mechanisms underlying the miRNA-disease associations still cannot be fully understood. In recent years, investigating the role of miRNAs in pathogenesis of human diseases has become one of the hottest topics [8], especially for entries from circulating miRNAs, epigenetics, miRNA-target interactions and genetics, whose number recorded in the HMDD has increased remarkably. As for interactions between miRNAs and targeted genes, for example, miRTar [27], an integrated system, identifies miRNA-target interactions in various scenarios and analyzes miRNA-targeted genes in pathways. In order to improve the accuracy of miRNA-gene target interaction identification, Pio et al. [28] presented a semi-supervised ensemble-based classifier that combines the prediction scores returned by several base algorithms to infer miRNA-targeted genes. They also predicted miRNA regulatory networks by a bi-clustering algorithm, which analyzes miRNA-target interactions to obtain inference results. The predicted miRNA-target interactions and miRNA regulatory network are stored in Co-clustered miRNA Regulatory Networks (ComiRNet) database. All of these researchers have shown that the miRNA regulatory network is complicated. Therefore, Chen et al. [29] developed a method to predict multiple types of miRNA-disease associations by a restricted Boltzmann machine (RBM) model. They constructed RBMs for miRNAs based on the data derived from HMDD v2.0, which included four types of miRNA-disease associations. Based on a contrastive divergence (CD) algorithm, they trained the constructed RBMs by initially setting the visible layer and hidden layer to obtain parameters of the RBM model. Finally, novel disease-related miRNAs and their types of interaction can be predicted by the trained RBM model. Although this method builds on the first model to predict multiple types of miRNA-disease associations, it only takes advantage of the data of known four types of miRNA-disease associations and ignores the relationships of disease-disease pairs and miRNA-miRNA pairs. Besides, RBM is a deep learning model and its training is time-consuming. In addition, the interaction prediction method among other types of biological entities can provide constructive suggestions for us in miRNA-disease interaction inference. By comparing over thirty network inference methods, Marbach et al. [30] observed that community-based methods can result in a powerful and robust performance for gene regulatory network reconstruction across different gold standards datasets. Therefore, Ceci et al. [31] proposed a semi-supervised method to deal with the problem of gene network reconstruction based on a multi-view learning framework. After assigning labels and identifying the multiple views, the method builds a classifier for each view, and then combines the output results of views to obtain final results. By applying a clustering algorithm, such as principle components analysis (PCA) or *k*-means, the views can be automatically identified by the system. This algorithm resolves the low quality and small quantity problem of known gene-gene interaction data and combines advantages of existing methods to achieve a good performance.

In this paper, a semi-supervised model called network-based label propagation method for inferring multiple types of miRNA-disease associations (NLPMMDA) is proposed by mutual information derived from the heterogeneous network. Label propagation is an efficient algorithm which can make full use of the information of labeled and unlabeled data and has been used in many studies [32,33,34,35]. A key of the NLPMMDA method is to construct a heterogeneous network. Firstly, a disease similarity homo-network is established by disease semantic similarity and Gaussian interaction profile kernel similarity. Secondly, a miRNA similarity homo-network is constructed in a similar way, which combines miRNA functional similarity and Gaussian interaction profile kernel similarity. Thirdly, a multi-type miRNA-disease association hetero-network is established by validating four types of miRNA-disease associations. Then NLPMMDA performs label propagation in each homo-network. The homo-networks are used to capture cluster structure among diseases and miRNAs, and the hetero-network is used to capture mutual information of miRNA and disease pairs. Finally, final label scores of miRNA-disease pairs under four types can be calculated by propagating information on the heterogeneous network. The results of LOOCV and case studies demonstrated the reliable performance of NLPMMDA.

## 2. Materials and Methods 

### 2.1. Data Preparation

In this paper, four types of human miRNA-disease association data were retrieved from HMDD [36]. In the updated database, human miRNA-disease data were annotated in four types, including entries from miRNA-target interactions, circulation samples, epigenetics and genetics [8]. After mapping the different miRNA precursors to mature miRNAs, the repeating miRNA-disease associations were removed. Finally, 682 miRNA-disease association data were obtained from miRNA-target interactions, 443 entries from circulations, 199 entries from epigenetics and 356 entries from genetics. All of these 1680 miRNA-disease associations are involved in 324 miRNAs and 171 diseases. These four types of miRNA-disease associations were used to construct a multi-type miRNA-disease association hetero-network which can offer the mutual interaction information. Besides, these four types of miRNA-disease associations are used as the gold standard dataset to evaluate the performance of our algorithm.

### 2.2. Construct Disease Similarity Homo-Network

The relationship of diseases can be represented by a directed acyclic graph (DAG) according to the disease classification system in the Medical Subject Headings (MeSH) database, in which nodes represent diseases and links represent the relationship of two diseases. For instance, a DAG of a disease $d_{i}$ can be represented as $DAG(d_{i})=(d_{i},V(d_{i}),E(d_{i}))$, where $V(d_{i})$ represents the vertices set of all ancestor diseases of $d_{i}$ and disease $d_{i}$ itself, and $E(d_{i})$ represents the edges set of corresponding links. According to the algorithm proposed in [18], semantic similarity value SS of $d_{i}$ and $d_{j}$ can be calculated by:

(1) $$SS(d_{i},d_{j})=\frac{\sum_{d\in V(d_{i})\;\cap \;V(d_{j})}\left(D_{d_{i}}(d)+D_{d_{j}}(d)\right)}{SV(d_{i})+SV(d_{j})},$$

where $D_{d_{i}}(d)$ is the contribution of disease d to the semantic value of disease $d_{i}$, the contribution of disease $d_{i}$ itself to its own semantic value is defined as 1 and the contribution of other diseases is defined as $max\left\{\Delta *D_{d_{i}}(d^{\prime })|d^{\prime }\in \mathrm{children}\;\mathrm{node}\;\mathrm{of}\;d\right\}$. Here, Δ is the semantic contribution factor to distinguish the different semantic contribution values of disease d in different layers of $DAG(d_{i})$; $SV(d_{i})$ is the semantic value of disease $d_{i}$, which can be defined as $\sum_{d\in V(d_{i})}D_{d_{i}}(d)$.

Gaussian interaction profile kernel similarity for diseases can be calculated by Gaussian kernel [37]. The miRNA interaction profile of a disease $d_{i}$ is defined as $DIP(d_{i})$, which is a binary vector to represent whether the disease $d_{i}$ interacts with every miRNA in the multi-type miRNA-disease association hetero-network. Thus, the Gaussian interaction profile kernel similarity $GS_{d}$ of disease $d_{i}$ and disease $d_{j}$ is defined as:

(2) $$GS_{d}(d_{i},d_{j})=\exp(-\gamma_{d}{\left\|DIP(d_{i})-DIP(d_{j})\right\|}^{2}),$$

where $\gamma_{d}$ is a parameter used to control the kernel bandwidth, which is set as $1/(\sum_{i=1}^{n_{d}}DIP{\left(d_{i}\right)}^{2}/n_{d})$. Here, $n_{d}$ is the total number of diseases.

By integrating the disease sematic similarity matrix and Gaussian interaction profile kernel similarity matrix for diseases, disease similarity matrix $S_{d}$ of disease similarity homo-network can be obtained as Equation (3).

(3) $$S_{d}(d_{i},d_{j})=\left\{ \begin{array}{l} SS(d_{i},\;d_{j}),d_{i}\;\mathrm{and}\;d_{j}\;\mathrm{have}\;\mathrm{semantic}\;\mathrm{similarity},\\ GS(d_{i},d_{j}),\;\mathrm{otherwise}. \end{array}\right.$$

In the disease similarity homo-network, the transition probability matrix is defined as:

(4) $$P_{d}=D_{d}^{-\frac{1}{2}}S_{d}D_{d}^{-\frac{1}{2}},$$

where $D_{d}$ is a diagonal matrix and $D_{d}(i,i)=\sum_{j\in N_{d}}S_{d}(i,j)$, and $N_{d}$ is the neighboring nodes set of disease *d.*

### 2.3. Construction of the miRNA Similarity Homo-Network

Similar to the construction of the disease similarity homo-network, the miRNA similarity homo-network is constructed based on miRNA functional similarity and Gaussian interaction profile kernel similarity. MiRNA functional similarity was calculated in a previous study [18]. The miRNA functional similarity value of miRNA $m_{i}$ and $m_{j}$ can be represented by $MFS(m_{i},m_{j})$. In order to reveal associations of miRNAs and diseases under different types, $MFS(m_{i},m_{j})$ is simply extended to multiple types of miRNA functional similarity matrix $MMFS(m_{i},m_{j},k)$, it is defined as:

(5) $$MMFS(m_{i},m_{j},k)=MFS(m_{｢i/n_{k}⎤},m_{｢j/n_{k}⎤}),$$

where k is the specific type, $n_{k}$ is the total number of types.

The Gaussian interaction profile kernel similarity matrix for miRNAs can be calculated by:

(6) $$GS_{m,k}(m_{i},m_{j})=\exp(-\gamma_{m,k}{\left\|MIP_{k}(m_{i})-MIP_{k}(m_{j})\right\|}^{2}),$$

where $MIP_{k}(m_{i})$ is a binary vector which can represent relationships of miRNA $m_{i}$ and the whole diseases under type k. $\gamma_{m,k}$ is a parameter used to control the kernel bandwidth which is set as $1/({\sum_{i=1}^{n_{m}}MIP_{k}(m_{i})}^{2}/n_{m})$. Here, $n_{m}$ is the number of miRNAs.

The integrated miRNA similarity homo-network is constructed:

(7) $$S_{m,k}(m_{i},m_{j})=\left\{ \begin{array}{ll} MMFS(m_{i},m_{j},k), & m_{i}\;\mathrm{and}\;m_{j}\;\mathrm{have}\;\mathrm{functional}\;\mathrm{similarity},\\ GS_{m,k}(m_{i},m_{j}), & \mathrm{otherwise}. \end{array}\right.$$

In the miRNA similarity homo-network, the transition probability matrix is defined as:

(8) $$P_{m,k}=D_{m,k}^{-\frac{1}{2}}S_{m,k}D_{m,k}^{-\frac{1}{2}},$$

where $D_{m,k}$ is a diagonal matrix and $D_{m,k}(i,i)=\sum_{j,k\in N_{m}}S_{m,k}(i,j,k)$, and $N_{m}$ is the neighboring nodes set of the miRNA *m* in miRNA homo-network.

### 2.4. Construction of the Multi-Type miRNA-Disease Association Hetero-Network

**Figure 1:**
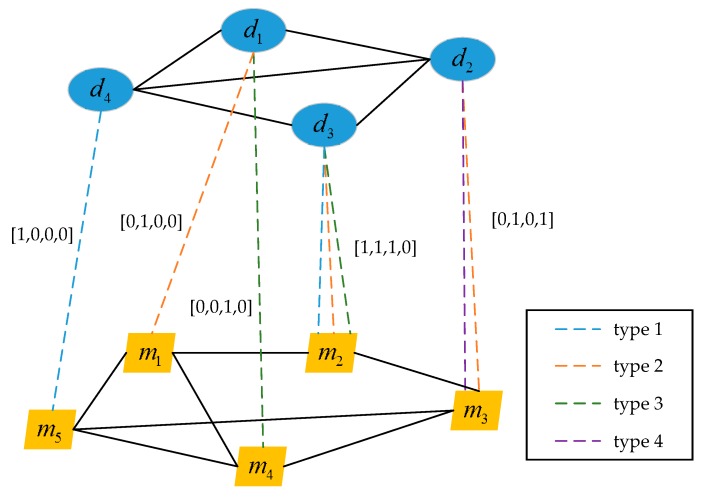
An example of the heterogeneous network composed of disease similarity homo-network, microRNAs (miRNA) similarity homo-network and multi-type miRNA-disease association hetero-network.

The multi-type miRNA-disease association hetero-network shows the relationships between miRNAs and diseases extracted from HMDD, including four types of human miRNA-disease association data. Figure 1 shows an example of the heterogeneous network, which contains four diseases and five miRNAs. The edges of multi-type miRNA-disease association hetero-network are created by four known types of miRNA-disease associations, and there are four edges between a disease and a miRNA at most. The edge vector $E_{ij}=\left\{e_{k}\right\}$ is used to represent the edges between disease $d_{i}$ and miRNA $m_{j}$, where $e_{k}=1$ if $d_{i}$ and $m_{j}$ has an association of type *k*, and $e_{k}=0$ otherwise. For example, if there are three association types between $d_{3}$ and $m_{2}$, then the edge vector is $E_{32}=[1,1,1,0]$. Based on the edge vectors, the adjacency matrix of multi-type miRNA-disease association hetero-network can be created. If disease $d_{i}$ and miRNA $m_{j}$ have confirmed associations, then $A(d_{i},m_{j})=E_{ij}$, where $i=1,\ldots ,n_{d}$, $j=1,\ldots ,n_{m}$, $n_{d}$ and $n_{m}$ are the number of diseases and miRNAs, respectively. 

Then, transition probability of miRNAs and diseases in hetero-network can be calculated by:

(9) $$P_{d,m,k}=D_{d,m,k}{}^{-\frac{1}{2}}AD_{d,m,k}{}^{-\frac{1}{2}},$$

where $D_{d,m,k}$ is a diagonal matrix and $D_{d,m,k}(i,i)=\sum_{j,k}A(i,j,k)$.

### 2.5. Network-Based Label Propagation Algorithm for Predicting Multiple miRNA-Disease Associations 

**Figure 2:**
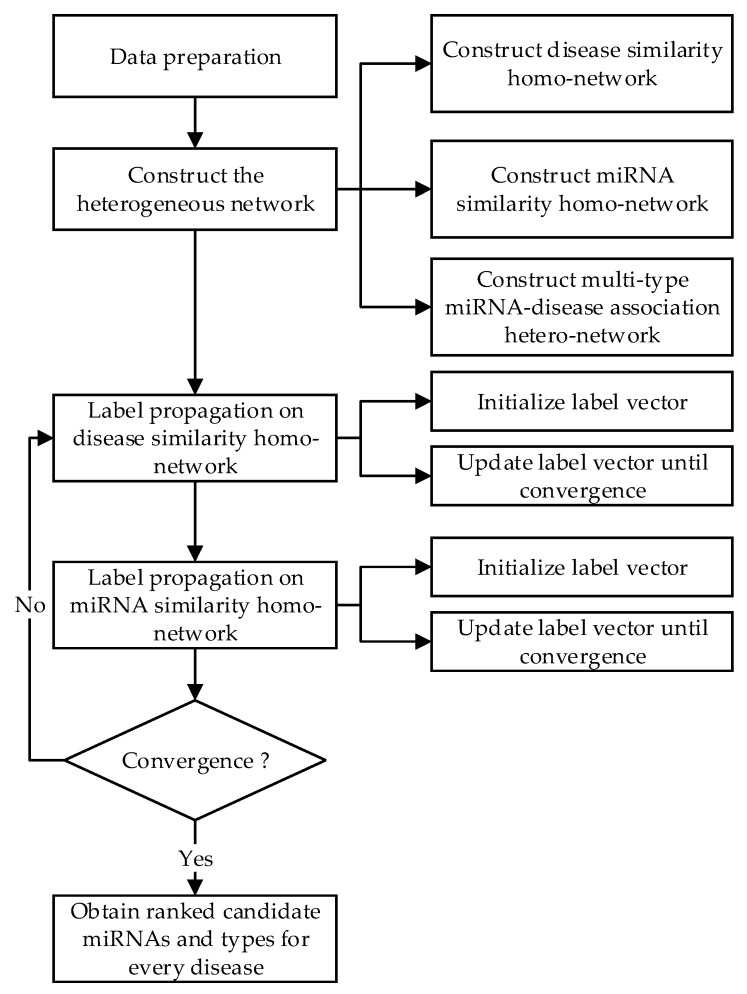
Flowchart of the Network-based Label Propagation Algorithm for Predicting Multiple miRNA-Disease Association (NLPMMDA).

Label propagation is a semi-supervised method. Its main purpose is to predict the labels of unlabeled data from both labeled and unlabeled data. A regularization framework for performing label propagation algorithm for a single network has been introduced and its convergence has been proved [35]. In this paper, label propagation is extended on a single network to our heterogeneous network, which is motivated by literature [38], and NLPMMDA is presented. Figure 2 shows the procedures of the NLPMMDA algorithm. The NLPMMDA method takes full advantage of mutual information in the heterogeneous network. Based on this method, novel disease-related miRNAs and the specific association types can be predicted.

The NLPMMDA algorithm can be described in detail as follows:

Step 1. Obtaining four types of miRNA-disease association data from HMDD and carrying out a data cleaning process.

Step 2. According to Section 2.2, Section 2.3 and Section 2.4, the heterogeneous network is constructed. In this study, the heterogeneous network G=(V,E) is composed of the disease similarity homo-network $G_{d}=(V_{d},E_{d})$, miRNA similarity homo-network $G_{m,k}=(V_{m,k},E_{m,k})$ and multi-type miRNA-disease association hetero-network $G_{d,m,k}=(V_{d}\cup V_{m,k},E_{d,m,k})$.

Step 3. Performing network-based label propagation algorithm on the disease similarity homo-network. For a given query disease, the final label vector can be obtained by iteratively implementing Equation (10).

(10) $$f_{d}^{t}=(1-\lambda_{d})f_{d}^{0}+\lambda_{d}P_{d}f_{d}^{t-1},$$

where $P_{d}$ is the transition probability matrix calculated by Equation (4); $f_{d}^{t-1}$ is a current label vector of diseases in which the ith element provides a current label score of disease $d_{i}$ at time t−1; $f_{d}^{t}$ is the final label vector of diseases; $f_{d}^{0}$ is the initial label vector of disease nodes, and it can be obtained by Equation (11).

(11) $$f_{d}^{0}=\frac{1-2\lambda_{d}}{1-\lambda_{d}}l_{d}^{0}+\frac{\lambda_{d}}{1-\lambda_{d}}P_{d,m,k}f_{m},$$

where $l_{d}^{0}$ is the current label vector of diseases which is derived from miRNA-disease interaction hetero-network; $f_{m}$ is the current label vector of miRNA nodes, $\lambda_{d}$ is a diffusion parameter of disease similarity homo-network which specifies the relative amount of information from its neighbors and its initial label; $P_{d,m,k}$ is the transition probability matrix calculated by Equation (9). Finally, $f_{d}^{t}$ converged to its limit $f_{d}$ when $\left\|f_{d}^{t}-f_{d}^{t-1}\right\|<\sigma$, where σ is a threshold to control terminate iteration.

Step 4. Performing network-based label propagation algorithm on the miRNA similarity homo-network to obtain the final label vector according to Equation (12).

(12) $$f_{m,k}^{t}=(1-\lambda_{m})f_{m,k}^{0}+\lambda_{m}P_{m,k}f_{m,k}^{t-1},$$

where $P_{m,k}$ is the transition probability matrix calculated by Equation (8); $f_{m,k}^{t-1}$ is the current label vector of miRNAs at time t−1; $f_{m,k}^{t}$ is the final label vector of miRNAs; $f_{m,k}^{0}$ is the initial label vector of miRNAs in four types, which is calculated by Equation (13).

(13) $$f_{m,k}^{0}=\frac{1-2\lambda_{m}}{1-\lambda_{m}}l_{m,k}^{0}+\frac{\lambda_{m}}{1-\lambda_{m}}P_{d,m,k}f_{d}^{},$$

where $l_{m}^{0}$ is the current label vector of miRNAs in which the jth element represents the current label score of miRNA $m_{j}$ under type k; $f_{d}^{}$ is the current label vector of diseases; $\lambda_{m}$ is a diffusion parameter of miRNA similarity homo-network. Similarly, the condition of convergence is $\left\|f_{m,k}^{t}-f_{m,k}^{t-1}\right\|<\sigma$, where σ is a threshold to control terminate iteration.

Step 5. Sequentially implementing network-based label propagation in the disease similarity homo-network and miRNA similarity homo-network to update the final label vector $f_{m}$ and $f_{d}^{}$ until both homo-networks converge. The condition of convergence is the same as mentioned above. Finally, for a given miRNA-disease pair, its final confidence label score in four types can be obtained. By ranking the label score in the final label vector, the top miRNAs are as considered as the most probable disease-related miRNAs and their type is considered as the most probable type.

## 3. Results

### 3.1. Performance Evaluation

**Figure 3:**
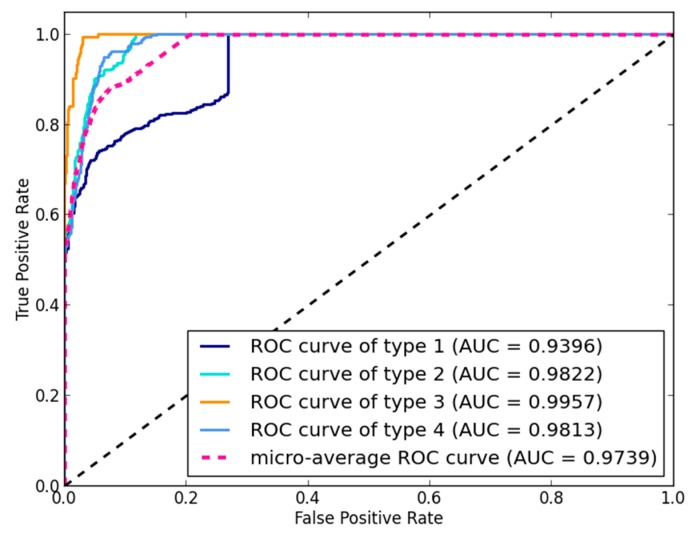
Receiver-operating characteristic (ROC) curve and area under the ROC curve (AUC) value of NLPMMDA based on leave-one-out cross validation (LOOCV). The micro-average AUC value of NLPMMDA is 0.9739. The AUC value of type 1, 2, 3, and 4 is 0.9396, 0.9822, 0.9957 and 0.9813, respectively.

In this study, to evaluate the performance of NLPMMDA, a LOOCV was implemented on four known and experimentally verified types of human miRNA-disease associations. Each known miRNA-disease association was left out in turn, and the remaining miRNA-disease associations were used as the labeled set. Then, the NLPMMDA method was implemented and the predictive scores of four types for each known miRNA-disease association were obtained. In addition, a receiver-operating characteristic (ROC) curve was drawn, which plots the true positive rate (TPR) versus the false positive rate (FPR) at different thresholds. The corresponding area under the ROC curve (AUC) was calculated to evaluate the predictive performance of the NLPMMDA method, where AUC = 1 means perfect performance and AUC = 0.5 means random performance. The ROC curve is typically used in binary classification problems to demonstrate the performance of a classifier. If a dataset only has positive and unlabeled samples, the ROC curve and AUC can be obtained by the ranked result of test samples. For example, in LOOCV, the test sample is ranked by the prediction scores of candidate miRNAs without confirmed association with currently investigated disease. In this paper, because the dataset can be divided into four classes, the output is operated by binarization and an ROC curve for each type is drawn. Finally, by considering each element of predictive scores as a binary prediction, the micro-average ROC curve was obtained. As can be seen in Figure 3, NLPMMDA obtained a reliable micro-average AUC value of 0.9739. The AUC value of four types of miRNA-disease associations is 0.9396, 0.9822, 0.9957 and 0.9813, respectively; type 1 represents entries from miRNA-target interactions, type 2 represents entries from circulation samples, type 3 represents entries from epigenetics and type 4 represents entries from genetics.

**Figure 4:**
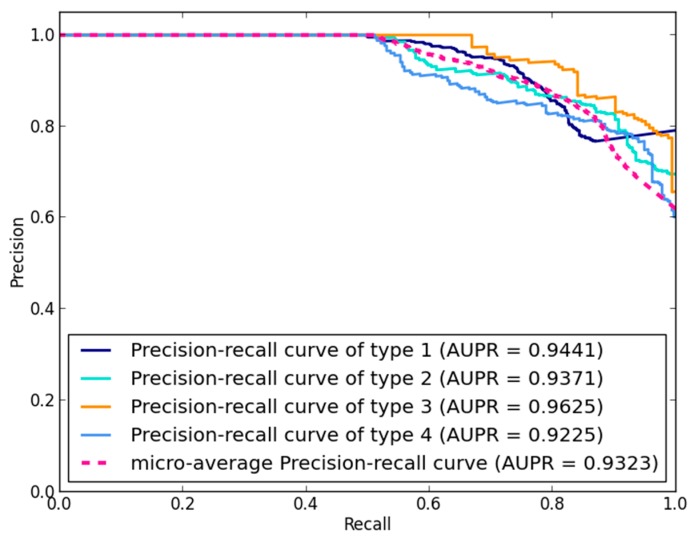
Precision-recall (PR) curve and area under the precision-recall (AUPR) value of NLPMMDA based on LOOCV. The micro-average AUPR value of NLPMMDA is 0.9323. The AUC value of type 1, 2, 3, 4 is 0.9441, 0.9371, 0.9625 and 0.9225, respectively.

Besides, considering the limited number of known miRNA-disease associations, the area under the precision-recall (AUPR) curve is applied to further evaluate the performance of NLPMMDA. The precision-recall (PR) curve plots the relationship between precision and recall at different thresholds, where high precision is related to a low false positive rate, and high recall is related to a low false negative rate. Generally, an AUPR value closer to 1 means the performance is better. As shown in Figure 4, the micro-average AUPR value of NLPMMDA is 0.9323, and the AUPR value for every type is 0.9441, 0.9371, 0.9625 and 0.9225, respectively.

### 3.2. Comparison with the Restricted Boltzmann Machine Model for Predicting Multiple Types of miRNA-Disease Associations Method

As far as we know, the restricted Boltzmann machine model for predicting multiple types of miRNA-disease associations (RBMMMDA) [29] is the first method to predict multiple types of miRNA-disease associations. It only makes use of known multiple types of miRNA-disease association data, and the AUC score of LOOCV is 0.8606. However, our method, NLPMMDA, integrates the information of disease semantic similarity, Gaussian interaction profile kernel similarity for diseases, miRNA functionally similarity, Gaussian interaction profile kernel similarity for miRNAs and the known four types of miRNA-disease associations, obtaining a better performance. The micro-average AUC value of NLPMMDA is 0.9739. Considering the complex structure of the RBM model, it is difficult to combine the disease similarity information and miRNA similarity information in the RBM model. The performances of RBMMMDA and NLPMMDA can be seen in Table 1. In addition, the RBM model has various parameters and parameter selection problem is not solved well, thus the parameters of the RBM model are simply a used experience value. Parameters of the NLPMMDA method are selected by the performance of the experiment. Besides, training of the RBM model takes a long time. However, NLPMMDA is a semi-supervised method, and the execution time is short.

### 3.3. Effect of the Parameters

There are two parameters $\lambda_{d}$ and $\lambda_{m}$ in the NLPMMDA algorithm. $\lambda_{d}$ is a diffusion parameter of disease similarity homo-network, which adjusts the relative amount of information from its initial label to its neighbors. $\lambda_{m}$ is a diffusion parameter of miRNA similarity homo-network. In this paper, $\lambda_{d}$ and $\lambda_{m}$ are set to the same value. By selecting different $\lambda_{d}$ and $\lambda_{m}$ values (varying from 0.1 to 0.9 with scale 0.1), LOOCV is implemented to obtain the AUC score of the NLPMMDA method. The LOOCV results are shown in Table 2. As a result, the AUC value is almost equal in the range of $0.1\leq \lambda_{d}\leq 0.4$ and $0.1\leq \lambda_{m}\leq 0.4$, and AUC value is decreased in the range of $0.6\leq \lambda_{d}\leq 0.9$ and $0.6\leq \lambda_{m}\leq 0.9$. However, our predictive method has no predictive ability when $\lambda_{d}$ and $\lambda_{m}$ are equal to 0.5, which is a result of the approach of initialization in homo-networks. Therefore, in this study, $\lambda_{d}=0.2$ and $\lambda_{m}=0.2$ are selected to predict novel miRNA-disease association types by the NLPMMDA algorithm. The optimal values of parameters depend on the known miRNA-disease association dataset.

### 3.4. Case Studies of Lung Cancer and Breast Cancer

To further confirm the robustness of the NLPMMDA method, case studies of lung cancer and breast cancer were implemented to evaluate the ability of the NLPMMDA method for predicting multi-types of miRNA-disease associations. All known miRNA-disease associations under four types were assigned as labeled data, and unknown miRNA-disease pairs were used as unlabeled data. Then, based on labeled and unlabeled data, NLPMMDA can predict miRNA-disease relationships and their specific types. Prediction results were manually verified by online databases and recent literature. The top 50 potential miRNA-disease association types of lung cancer and breast cancer are listed in Table 3 and Table 4, respectively, including disease-related miRNAs, miRNA-disease association types and evidences related to miRNA-disease pairs. The evidence is the PubMed Unique Identifier (PMID) of related literature. Due to the complexity of diseases and the associated miRNA roles, a predicted association type supported by three PubMed articles at least can be considered as a reliable association type.

The morbidity and mortality of lung cancer is high in both men and women, and lung cancer is the most common cause of cancer death worldwide [39]. Although various new therapeutics and strategies for detection and early diagnosis have progressed in lung cancer, its prognosis remains poor [40]. Recent studies demonstrated the important role of miRNAs in development and therapy response of lung cancer. In the labeled data, there are 52 miRNA-disease associations, which are classified as the miRNA-target type [41,42], circulating miRNA type [43,44], epigenetics type [45] and genetics type [46,47]. After implementing the NLPMMDA method on labeled and unlabeled data, scores of miRNA-disease pairs are predicted. As a result, among top 20 and top 50 candidates without relevance of known association types, 17 and 44 lung cancer-related miRNAs and their association types are supported by different evidence, respectively, and 25 predicted results are considered as reliable association types. As shown in Table 3, in the top 50 potential lung cancer-related miRNAs, miR-133a plays a tumor suppressor role in non-small cell lung cancer (NSCLC) by targeting *IGF-1R*, *TGFBR1* and *EGFR* [48]. Also, in NSCLC, miR-143 targets *ATG2B* and miR-34a targets *TGFβR2* to inhibit cell proliferation [49,50]; Besides, serum miR-126 and miR-21 levels can be used as novel biomarkers in non-small cell lung cancer development, metastasis and screening [51,52], and circulating miR-29a shows a highly prognostic signature in non-squamous NSCLC patients [53]. The single nucleotide polymorphisms rs2910164 of miR-146a are associated with the risk of NSCLC in the Chinese population, which can be regarded as the genetics type [54].

Based on annual statistical data, breast cancer is one of the most common types of cancer which mainly occurs in women [55]. Current studies demonstrated related death rates of breast cancer are still on the rise [56]. Besides, accumulating evidence shows that miRNAs play a vital role in breast cancer and can be used as diagnosis and therapeutic biomarkers for breast cancer patients. In our labeled data, there are 176 known miRNAs-disease associations which can be divided into four types according to evidence from literature. For example, serum miR-155 is up-regulated in breast cancer patients; thus, serum miR-155 is a potential biomarker to track breast cancer [57,58]. According to HMDD, the association between miR-155 and breast cancer is labeled as the circulation type [8]. The candidate miRNAs without known breast cancer-related miRNAs and their association types are predictive by the NLPMMDA method. Among the top 20 and top 50 potential miRNAs, 17 and 37 miRNA-disease association types are confirmed by biological evidence, respectively, and 16 predicted results are considered as reliable association types. Table 4 shows the details. Hsa-miR-1 is a breast cancer-related miRNA in the HMDD database. However, their underlying association type is not clear. In our predictive result, the relationship between hsa-miR-1 and breast cancer is target type, which can be proved by various evidence. For example, as described in the result of Liu et al. [59], hsa-miR-1 can function as a tumor suppressor in breast cancer by targeting *K-RAS* and *MALAT1*. Also, *IMPDH1* and *NPEPL1* genes are identified as direct targets of miR-19a in breast cancer by a quantitative proteomic strategy [60]; miR-19b can promote metastasis of breast cancer by targeting *MYLIP* and its related cell adhesion molecules [61]; and miR-133a acts as a tumor suppressor in breast cancer by targeting *EGFR* [62]. Moreover, the plasma level of circulating miR-146a is involved in breast cancer biology and tumor progression [63]. In primary human breast cancer, hsa-miR-9 is affected by epigenetic inactivation because of aberrant hypermethylation [64].

In conclusion, 44 and 37 out of the top 50 predictive lung cancer-related and breast cancer-related miRNAs and their specific association types are confirmed by experimental evidence, respectively. The results of case studies demonstrated the robustness of NLPMMDA method.

### 3.5. Web Server for Network-Based Label Propagation Algorithm to Predicting Multiple miRNA-Disease Association Method

In this study, a web server was built to show the prediction results of the NLPMMDA method, which is freely available at http://39.107.230.144/NLPMMDA.

The web server enables the function of predicting four types of miRNA-disease associations based on the NLPMMDA algorithm. The final prediction result for a specific disease will be shown in a table, and the rank, miRNA name, association type and potential association probability will be included. The tables contain known verified related miRNAs and types for a disease, whose value of potential association probability is 1.0.

## 4. Discussion

Increasing evidence indicates the prominent role of miRNAs in the development of various diseases. Understanding the underlying mechanisms of miRNAs in diseases is becoming an urgent problem worldwide. In this study, a network-based label propagation algorithm is proposed to infer specific types of miRNA-disease associations, which integrated four types of known human miRNA-disease associations derived from HMDD. The NLPMMDA method constructed a heterogeneous network, in which a disease similarity homo-network is constructed by integrating disease sematic similarity information with Gaussian interaction profile kernel similarity information, and miRNA similarity homo-network is constructed by integrating miRNA functional similarity information with Gaussian interaction profile kernel similarity information. Besides, a multi-type miRNA-disease interaction hetero-network is constructed by four types of known miRNA-disease association data. In addition, the traditional label propagation algorithm is extended to the heterogeneous network and the strategy of label initialization is changed in the NLPMMDA method. The LOOCV result, case studies of lung cancer, and breast cancer demonstrate the reliable performance of the NLPMMDA method.

Compared with current computational methods which can predict multiple type miRNA-disease associations, the NLPMMDA method achieves a better performance because of several factors. Firstly, the network-based label propagation algorithm is a semi-supervised machine learning model. As we all know, one of the current difficulties of predictive models is the selection of negative samples. NLPMMDA does not require verified negative miRNA-disease associations. Secondly, transition probability among diseases and miRNAs under four types are calculated in the NLPMMDA method, which can capture the similarity information from neighboring nodes in homo-networks and improve the predictive function of the computational model. Thirdly, construction of heterogeneous network could offer mutual information between the miRNA similarity homo-network and disease homo-network. The label values of nodes in the homo-networks are initialized by their initial labels and neighbors from other homo-networks, which makes label confidence score more reliable. Although NLPMMDA exhibited highly reliable results, it still has some limitations. Transition probability scores among four types are simply calculated by miRNA functional similarity and Gaussian interaction profile kernel similarity, which may result in offset error. In addition, the NLPMMDA method is not applicable to diseases without any known associations of miRNAs. The different combination of diffusion parameters in homo-networks may improve the performance of the NLPMMDA method, which can be further studied in the future.

## Figures and Tables

**Table 1:** Comparison with the restricted Boltzmann machine model for predicting multiple types of miRNA-disease associations (RBMMMDA) method.

Algorithms	RBMMMDA	NLPMMDA
AUC	0.8606	0.9739
Data	Known four types of miRNA-disease associations	Disease semantic similarity, miRNA functional similarity, Gaussian interaction profile kernel similarity and known four types of miRNA-disease associations
Application	Cannot be applied to isolated diseases	Cannot be applied to isolated diseases
Parameters	Use the previous value	Select by the performance of experiments
model	Supervised learning	Semi-supervised learning
Case study	Lung cancer: 33 of top 50	Lung cancer: 44 of top 50
	Breast cancer: 17 of top 50	Breast cancer: 37 of top 50

**Table 2:** Effect of the parameters.

$\lambda_{d}$	$\lambda_{m}$	AUC	AUPR
0.1	0.1	0.9738	0.9320
0.2	0.2	0.9739	0.9323
0.3	0.3	0.9738	0.9309
0.4	0.4	0.9720	0.9302
0.5	0.5	0.5	0.5
0.6	0.6	0.8173	0.6490
0.7	0.7	0.8076	0.6409
0.8	0.8	0.7900	0.6251
0.9	0.9	0.7559	0.5962

**Table 3:** Lung cancer-related candidate miRNAs and association types predicted by NLPMMDA.

miRNAs	Types	PMID	miRNAs	Types	PMID
hsa-mir-499a	genetics	unconfirmed	hsa-mir-19a	target	27588137;25604748;28592790
hsa-mir-146a	genetics	25154761;24144839;29127520	hsa-let-7f	target	29017393
hsa-mir-133a	target	24816813;22089643;25518741	hsa-mir-15a	target	25442346;24500260;25874488
hsa-mir-126	circulation	28253725;27093275;29266846	hsa-mir-206	target	26919096;26075299;25522678
hsa-mir-17	genetics	17384677	hsa-mir-16	genetics	unconfirmed
hsa-mir-21	circulation	25501703;25421010;29163821	hsa-mir-126	target	18602365;22510476;29277611
hsa-mir-143	target	25322940;25003638;24070896	hsa-mir-125b	target	28713974
hsa-mir-34a	target	25501507;25038915;24983493	hsa-mir-218	target	21159652;24247270;24705471
hsa-mir-20a	genetics	17384677	hsa-mir-17	circulation	23263848
hsa-mir-29a	circulation	24928469	hsa-let-7e	target	unconfirmed
hsa-mir-200c	target	24997798;24205206;23708087	hsa-mir-20a	target	24722426
hsa-mir-17	target	24755562;24722426;29289833	hsa-mir-219	target	28714014
hsa-mir-92a	genetics	unconfirmed	hsa-mir-222	target	21042732
hsa-mir-20a	circulation	25421010	hsa-mir-19b	target	28364280
hsa-mir-34a	epigenetics	18719384	hsa-mir-429	target	24866238;27602157
hsa-mir-34b	epigenetics	24130071;22047961;21383543	hsa-mir-223	circulation	28356944;25421010;29212284
hsa-mir-18a	genetics	unconfirmed	hsa-mir-18a	target	28471447
hsa-mir-200b	target	22139708 ;28731781;28615992	hsa-mir-122	circulation	24282590;25926378
hsa-mir-155	target	22027557 ;29260515;28939896	hsa-let-7a	target	21097396
hsa-mir-16	target	25435430;23954293;29138833	hsa-mir-15a	genetics	unconfirmed
hsa-mir-34c	epigenetics	24130071;22047961;21383543	hsa-mir-124	epigenetics	17308079
hsa-mir-221	target	18246122;21042732;19962668	hsa-mir-92a	target	23820254
hsa-mir-183	target	18840437;26951513;27593936	hsa-mir-133b	target	22883469;19654003;29328427
hsa-mir-214	target	28396596;26462018;28396596	hsa-mir-155	genetics	28225782
hsa-mir-146a	circulation	28678319;25755772;24531034	hsa-mir-203	target	25140799;24040137;28921827

PMID: PubMed Unique Identifier.

**Table 4:** Breast cancer-related candidate miRNAs and association types predicted by NLPMMDA.

miRNAs	Types	PMID	miRNAs	Types	PMID
hsa-mir-16	genetics	16754881;17012848	hsa-mir-127	target	24282530;24155205;25477702
hsa-mir-1	target	26275461;26926567;26497855	hsa-let-7i	target	24662829;21826373;
hsa-mir-126	circulation	28683441	hsa-let-7a	genetics	26681038
hsa-mir-19a	target	22952885;23831570;27596294	hsa-mir-106b	target	27519168;27325313;28518139
hsa-let-7a	target	24172884	hsa-mir-219	target	Unconfirmed
hsa-mir-19b	target	28969074;28731027;27602768	hsa-let-7f	genetics	23042301
hsa-mir-92a	genetics	Unconfirmed	hsa-mir-127	epigenetics	27998789
hsa-mir-223	circulation	Unconfirmed	hsa-mir-15b	target	25783158
hsa-mir-18a	target	19684618;25069832;21755340	hsa-mir-143	target	28746466;28559978;28588724;27121210
hsa-mir-29a	circulation	Unconfirmed	hsa-mir-19b	circulation	Unconfirmed
hsa-let-7c	target	25388283	hsa-mir-199a	circulation	26476723;25906045
hsa-mir-125b	genetics	19738052	hsa-let-7e	genetics	Unconfirmed
hsa-mir-133a	target	23786162;29207145;26107945	hsa-mir-145	circulation	23334650
hsa-mir-15a	target	27596816;27713175;28655885	hsa-mir-155	genetics	26095675
hsa-let-7d	target	22081076	hsa-let-7d	genetics	Unconfirmed
hsa-let-7f	target	22407818;25552929	hsa-mir-218	circulation	Unconfirmed
hsa-mir-29b	epigenetics	24297604	hsa-mir-221	circulation	25009660;22156446
hsa-mir-214	target	24577056;25738546;28071724	hsa-mir-146a	target	27175941;25596948;25712342
hsa-mir-9	epigenetics	26519551;17948228	hsa-mir-124	epigenetics	Unconfirmed
hsa-mir-146a	circulation	27197674;26033453;23898484	hsa-mir-19a	circulation	24938880;24416156
hsa-let-7e	target	Unconfirmed	hsa-let-7g	target	21868760
hsa-mir-18a	circulation	24694649;23705859;28109133	hsa-mir-106a	target	27325313
hsa-mir-25	target	25026296;29310680;28188287	hsa-mir-9	circulation	Unconfirmed
hsa-let-7b	target	21826373;24264599;23339187;22761738	hsa-mir-145	genetics	Unconfirmed
hsa-mir-92a	target	28881597;29162724;28881597	hsa-mir-19b	epigenetics	Unconfirmed
